# The impact of nutrition on tendon health and tendinopathy: a systematic review

**DOI:** 10.1080/15502783.2022.2104130

**Published:** 2022-08-03

**Authors:** Aveline Hijlkema, Caroline Roozenboom, Marco Mensink, Johannes Zwerver

**Affiliations:** a Wageningen University and Research, Division of Human Nutrition and Health, Wageningen, The Netherlands; b Research Support Center, Hospital Gelderse Vallei, Ede, The Netherlands; c Hospital Gelderse Vallei, Sports Valley, Department of Sports Medicine, Ede, The Netherlands; d University of Groningen, University Medical Center Groningen, Center for Human Movement Sciences, Groningen, The Netherlands

**Keywords:** Tendon, tendinopathy, diet, supplements, collagen

## Abstract

**Background:**

Tendinopathy is a painful condition that is prevalent in athletes as well as the general human population, and whose management is challenging.

**Objective:**

This systematic review aimed to evaluate the impact of nutrition on the prevention and treatment of tendinopathy.

**Methods:**

Searches were conducted in PubMed, EMBASE, Web of Science, and SPORTDiscus without restriction to year of publication. Studies examining the impact of exposure to nutrient intake in an adult human population on 1) prevalence/incidence of tendinopathy, 2) clinical outcomes of tendinopathy, 3) structural changes in the tendon by imaging modalities. Experimental and observational study designs written in English, Dutch, or German were eligible.

**Results:**

Nineteen studies met the inclusion criteria. The effects of the habitual diet were investigated in one study. Four studies examined the effects of exposure to alcohol. Alcohol consumption can be a potential risk factor associated with Achilles tendinopathy and rotator cuff tears, although findings were inconsistent. The use of dietary supplements was examined in fourteen studies. Among these, collagen-derived peptides were most often part of the supplements evaluated. Combining training and dietary supplements seems to induce better clinical and functional outcomes in tendinopathy.

**Conclusion:**

This review demonstrates the paucity of high-quality studies and a wide variety among studies regarding nutrients, tendon location, study population, and reported outcome measures. Individual studies showed promising clinical implications for the use of dietary supplements, particularly those containing collagen-derived peptides. However, giving any definitive dietary recommendations on the prevention and treatment of tendinopathy remains elusive.

## 1. Introduction

Tendinopathy, which involves persistent tendon pain and loss of function related to mechanical loading [1], is common in athletes as well as in the general population [2,3]. Mechanical overuse is seen as the key initial trigger in the multifactorial etiology of tendinopathy [3], hence it is a common cause of injury in sports that involve exposure to high forces and repetitive movements, such as running, volleyball, and tennis [3–5]. Tendinopathy is also prevalent in workers exposed to monotonous repetitive work tasks [2], and is associated with a number of medical conditions such as obesity and diabetes mellitus [6–8]. Other intrinsic risk factors are age, sex, and genetics [9]. The potential suffering from pain and loss of function may impact health, sports performance, and work ability [10]. Also, the impact of tendinopathy on quality of life is substantial, specifically on the domains mobility, pain/discomfort, and usual activities [11].

There is only limited evidence for the efficacy of preventive interventions for tendinopathy [12]. Numerous treatment options for tendinopathy have been described [9], but convincing evidence of success of many common therapies is lacking. Hence the management of tendinopathy remains a challenging and often time-consuming process [9]. This stresses the relevance of additional strategies for both prevention and treatment of tendinopathy.

The pathological tendon is characterized by an altered tissue homeostasis [13]. Given that diet plays a major role in the homeostasis of all tissues and poor nutrition is one of the extrinsic factors that contributes to the development of tendinopathy [9], nutritional interventions, e.g. intake of collagen, are a plausible, potential strategy to improve the prevention and healing of tendinopathy.

Adequate intake of nutrients – macronutrients as well as micronutrients – is of great importance, especially for populations with specific demands such as athletes. This primarily entails consumption of a healthy habitual diet, but also sport-specific nutritional strategies as well as dietary supplements may be used to optimize intake in specific situations. A dietary supplement is defined as a food, food component, nutrient, or non-food compound that is purposefully ingested in addition to the habitually consumed diet with the aim of achieving a specific health and/or performance benefit [14]. The use of supplements is widespread among athletes as well as the general population [15]. However, specific recommendations of dietary strategies for preventing or treating tendon injuries, either as part of the habitual diet or with additional dietary supplements, are lacking [16].

The potential benefits of nutrition on tendon health have been described in only a few reviews [17–19]. A recent short review identified various nutrients, including amino acids, vitamins, and trace minerals, as being potentially useful in improving tendon growth and healing [17]. It has additionally been suggested that nutritional interventions involving multiple nutrients, e.g. collagen combined with vitamin C, may be more effective than single-nutrient strategies, as many nutrients are involved in tendon and collagen metabolism [17]. So far, only evidence for effects of vitamin and amino acid supplements on tendon tissue healing has systematically been evaluated, but hardly any clinical studies are included [20,21]. No previous study has systematically synthesized the evidence of nutritional exposure for clinical outcomes on physical, psychosocial and overall life impact, or the risk of tendinopathies in relation to nutrition. The aim of this systematic review was therefore to evaluate the impact of nutrition on the prevention and treatment of tendinopathy in a general human population. This will guide future studies on directions of research toward evidence-based nutritional recommendations to prevent and treat tendinopathy, which ultimately leads to a lower prevalence and better management of this bothersome condition.

## 2. Methods

This systematic review complied with the PRISMA guidelines [22]. The study protocol was registered with the International Prospective Register of Systematic Reviews (PROSPERO) on 29 June 2020 (CRD42020189773).

### Eligibility criteria

Studies were eligible if they investigated the impact of exposure to nutrient intake (either as part of the habitual diet or in the form of specific dietary supplements) in an adult (>18 years) human population, using at least one of the following outcome measures: prevalence/incidence of tendinopathy, a clinical outcome that captures one of the tendinopathy-related core domains as established by the ICON group [23] (see Additional file 1), structural changes in the tendon identified by imaging modalities such as magnetic resonance imaging (MRI) or ultrasound imaging (USI) [24]. Randomized and non-randomized intervention studies and cohort, case-control, cross-sectional, and case studies written in English, Dutch, or German were included. Reviews, letters, and editorials were excluded. There was no restriction with regard to year of publication.

### Search strategy

We searched the electronic databases PubMed, EMBASE, Web of Science and SPORTDiscus in June 2020 for eligible studies. The specific search strategies were created by a health science librarian with expertise in systematic review searching and had three components: Nutrition, Tendinopathy, and Human. The PubMed search strategy was adapted to the syntax of other databases and is presented in Additional file 2. In addition to the database search, reference lists of included studies as well as relevant reviews were manually checked to identify additional studies for inclusion.

### Study selection

The records were imported into Endnote X9, where duplicates were removed. Two reviewers (AH, JZ) independently screened the titles and abstracts from the identified articles for eligibility, followed by full-text evaluation for final study inclusion. Any disagreements about inclusion/exclusion were discussed between the reviewers and a final decision was made by all authors.

### Data extraction and analysis

Data was extracted from the included papers using a spreadsheet prepared in Microsoft Excel. We extracted publication details, study design, study aim, population characteristics, type of tendinopathy/site of interest, exposure and comparator details, relevant outcome measures and results, and conclusion with respect to nutritional exposure. The study characteristics and results are presented in tables and summarized semi-narratively.

### Risk of bias assessment

Two reviewers (AH, CR) independently assessed risk of bias of the included studies. The revised Cochrane risk-of-bias tool (RoB 2) was used to assess the quality of randomized trials [25]. We assessed risk of bias on a per-protocol basis for all five domains: (1) randomization process, (2) deviations from intended interventions, (3) missing outcome data, (4) outcome measurement, and (5) selection of the reported result. Other intervention, cohort, case-control, and cross-sectional studies were assessed using the ROBINS-I tool [26]. Bias was assessed for the following domains: (1) confounding, (2) selection of participants into the study, (3) classification of interventions, (4) deviations from intended interventions, (5) missing data, (6) measurement of outcomes, and (7) selection of the reported result.

An overall level of certainty in the evidence for clinical outcomes and for the occurrence/prevalence of tendinopathy was rated using the GRADE approach for systematic reviews in which only a narrative summary of the effect across studies is available [27].

## 3. Results

**Figure 1.**
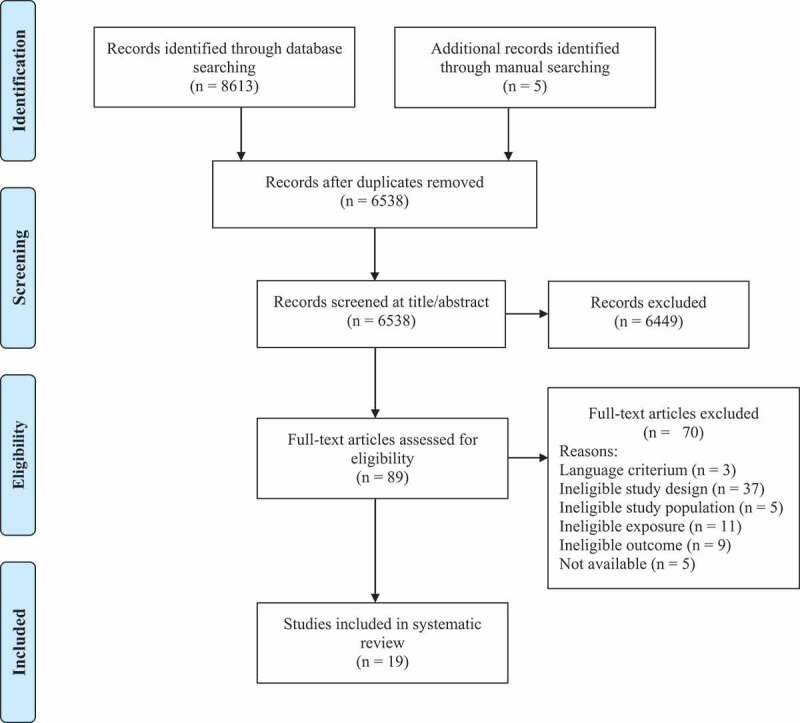
PRISMA flow diagram of the study selection process.

Figure 1 shows the study selection process. A total of 8618 records were retrieved from the database and manual searches. After duplicates were removed, 6538 records were screened for eligibility, of which 89 were assessed in full-text. Of these studies, nineteen met the inclusion criteria. Seventy articles were excluded because they did not meet the language (n = 3), study design (n = 37), study population (n = 5), exposure (n = 11) or outcome (n = 9) criteria, or were not available (n = 5). Details of the included studies are provided in Tables 1 and 2. Five studies investigated the effect of the habitual diet (Table 1). The use of dietary supplements was examined by fourteen studies (Table 2), one of which evaluated an intervention of supplement use combined with habitual dietary changes [28].

**Table 1.** Details of the studies examining exposure to the habitual diet (n = 5).

Study	Design	Aim	Population	Nutritional exposure	Outcome measure(s)	Results	Conclusion
Hjerrild et al. (2019) [31]	Cross-sectional study	To investigate the effects of life-long physical activity on skin autofluorescence (SAF) and AT structure, and to determine if SAF and tendon structure are influenced by dietary factors	182 athletes + 24 sedentary persons (54 ±18 y, male)	Diet (fruit, vegetables, fish, bread, cereals, coffee, wine, beer, liqueurs, total fluid, pure water) as well as overall dietary pattern (Western vs. Mediterranean) currently and during youth	Anteroposterior AT thickness (USI)	None of the dietary parameters was a significant predictor of AT thickness	Diet did not affect AT thickness
Jain et al. (2018) [29]	Prospective cohort study	To assess predictors of better shoulder pain and function after surgery	50 patients with symptomatic RC tears undergoing operative treatment (59 ±9 y, 62% male)	Alcohol (habitual consumption <2-3/month vs. >1-2/week)	Shoulder pain and function (SPADI) at 3, 6, 12 and 18 months follow-up	Those consuming alcohol >1-2 times/week had lower SPADI scores (less shoulder pain and better function) than those consuming alcohol <2-3 times/month (p = 0.017)	Alcohol use is a longitudinal predictor of pain and functional outcomes after operative treatment for RC tears
Owens et al. (2013) [30]	Prospective cohort study	To prospectively identify risk factors for the development of lower extremity tendinopathy and plantar fasciitis in United States military personnel	80,106 US active-duty military personnel (70.1% male)	Alcohol (none vs. light/moderate/heavy)	Risk of AT and PT tendinopathy (OR)	Moderate weekly alcohol consumption was marginally associated with increased risk for AT tendinopathy (OR = 1.33 (1.00-1.76), but not for PT tendinopathy (OR = 0.93 (0.71-1.21))	Alcohol consumption is a potentially modifiable risk factor associated with AT tendinopathy
Passaretti et al. (2016) [33]	Case-control study	To investigate the association between alcohol consumption and RC tears	249 patients treated arthroscopically for RC repair + 356 controls without RC tears (cases: 64 (54-78) y, 56% male; controls: 66 (58-82) y, 52% male)	Alcohol (nondrinkers vs. moderate/excessive drinkers)	Risk of RC tears (OR)	Significant risks of RC tears for excessive drinkers (men: OR = 1.7, p = 0.04; women: OR = 1.9, p = 0.04)	Long-term alcohol intake is a significant risk factor for onset and severity of rotator cuff tears
Rechardt et al. (2010) [32]	Cross-sectional study	To assess the associations of lifestyle factors, metabolic factors and carotid intima-media thickness with shoulder pain and chronic (>3 months) RC tendinitis.	6237 participants (male: 50.8 y; female: 52.9 y, 46% male)	Alcohol (none/light/moderate/heavy)	Risk of RC tendinitis (OR)	Alcohol consumption was not associated with chronic RC tendinitis in either gender (data not shown)	(no conclusion with regard to alcohol)

AT, Achilles tendon; CG, control group; OR, odds ratio; PT, patellar tendon; RC, rotator cuff; SPADI, Shoulder Pain and Disability Index; TG, treatment group; USI, ultrasound imaging

**Table 2.** Details of the studies examining exposure to dietary supplements (n = 14).

Study	Design	Aim	Population	Nutritional exposure	Concurrent exposure	Comparator	Outcome measure(s)	Results	Conclusion
Arquer et al. (2014) [44]	Non-comparative intervention study	To evaluate the efficacy and safety of a nutritional supplement on the clinical and structural evolution of AT, PT and LET tendinopathies	98 tendinopathy patients, AT (n = 32): 49.2 ±3.64 y; PT (n = 32): 47.7 ±1.69 y; LET (n = 34): 39.0 ±2.44 y, both sexes	3 capsules Tendoactive (mucopolysaccharides (435 mg), type I collagen (75 mg), vitamin C (60 mg)) per day for 90 consecutive days	None	Pre-measurements	Pain intensity at rest and when active (VAS); joint function (VISA-A/VISA-P/PRTEE); tendon cross-sectional thickness (USI)	After 90 days: Pain at rest decreased by 80% (AT), 71% (PT) and 91% (LET) (p <0.001). Pain when active decreased by 82% (AT), 73% (PT) and 81% (LET) (p <0.001). Functional scores improved by 38% (AT), 46% (PT) and 77% (LET) (p <0.001). Thickness reduced by 12% (AT), 10% (PT) and 20% (LET) (p <0.05).	Administration of Tendoactive is effective for improving the clinical symptoms and structural evolution of tendinopathies
Balius et al. (2016) [36]	RCT	To determine the additional benefit of mucopolysaccharides, collagen and vitamin C (MCVC) to a physical therapy program in patients with AT tendinopathy	58 reactive or degenerative AT tendinopathy patients (18-70 y, both sexes)	3 capsules MCVC (mucopolysaccharides (435 mg), type I collagen (75 mg), vitamin C (60 mg)) per day for 3 months	Eccentric training (EC+MCVC) or passive stretching (PS+MCVC)	Eccentric training only (EC)	VISA-A; pain at rest and during activity (VAS); tendon bilateral thickness (USI)	After 12 weeks: Statistically and clinically significant improvement in VISA-A scores in all groups without between-group effect (p >0.1). VAS scores decreased in all groups with a difference for pain at rest between PS+MCVC (−3.7(0.8) and EC (−2.7(1.3), p <0.05). Bilateral thickness remained constant in EC and EC+MCVC, and reduced in PS+MCVC (−0.63(0.3) mm, p <0.05).	MCVC seems to be therapeutically useful for the management of tendinopathies
Praet et al. (2019) [39]	RCT (cross-over)	To investigate whether oral supplementation of specific collagen peptides improves symptoms and tendon vascularization in patients with chronic mid-portion AT tendinopathy in combination with structured exercise	20 mid-portion AT tendinopathy patients (44 ±8 y, 65% male)	Two daily sachets Tendoforte (2.5 g hydrolyzed specific collagen peptides) for 3 months	Eccentric and running exercises for 6 months	Placebo + eccentric and running exercises for 6 months	Pain and functional limitations (VISA-A); vascularization (USI)	The group receiving the supplement in the first 3 months improved by 12.6 (9.7-15.5) in the supplemental phase and 5.9 (2.8-9.0) in the placebo phase. The other group improved by 5.3 (2.3-8.3) and 17.7 (14.6-20.7). There was a difference between groups in evolution of the VISA-A scores over time (p <0.0001). No difference in vascularization between groups.	Supplementation of specific collagen peptides may accelerate the clinical benefits of exercise program in AT patients.
Vitali (2019) [43]	Non-randomized controlled study	To determine the efficacy of Extracorporeal Shock Wave Therapy (ESWT) in combination with the dietary supplement Tendisulfur Forte in the treatment of shoulder, LET and AT tendinopathies	90 AT, shoulder or LET tendinopathies (39-69 y, 50% male)	Tendisulfur Forte (containing methyl-sulfonyl-methane (MSM), hydrolyzed swine collagen (Type I and Type II), L-arginine and L-lysine, vitamin C, chondroitin sulfate, glucosamine, Curcuma longa extracted to obtain curcuminoids, dry Boswellia serrata extracted to obtain acetyl-11-keto-b-boswellic acid (AKBA), and myrrh) 2x day for 1 month, 1x day for 1 month	ESWT	ESWT	Pain (VAS); clinical functional evaluation (VISA-A, UCLA shoulder score, MEPS)	After 60 days: UCLA scores were higher in TG [27] compared to CG (23, p = 0.0002). MEPS was higher in TG compared to CG (p <0.00001). VISA-A scores improved more in TG (+27, +39%) compared to CG (+7, 17%). VAS scores were lower in TG compared to CG for all tendinopathy types (p <0.0001).	Combined treatment of ESWT and oral supplementation leads to a faster recovery and better outcomes of AT, shoulder and LET tendinopathy.
Merolla et al. (2015) [35]	RCT	To assess the analgesic effect of a dietary supplement containing Boswellia serrata and Curcuma longa in a population of subjects with full-thickness SSP tendon tear treated arthroscopically	100 patients who underwent surgical SSP tendon repair (TG: 53.3 ±7.6 y, 54% male; CG: 55.4 ±9.4 y, 56% male)	Two daily sachets for 15 days, 1 sachet Tendisulfur (methyl-sulfonyl-methane, type I and II collagen, glycosaminoglycans, L-arginine, L-lysine, Boswellia serrata dry extract titrated to 30% inacetyl-1 1-keto-B-boswellic acid, Curcuma longa dry extract titrated to 95% curcuminoids) for 45 days	Conventional analgesic therapy	Placebo + conventional analgesic therapy	Overall pain, and pain at night, during activity and at rest (VAS); CMS; shoulder function (SST)	Lower overall and night pain scores in TG compared to CG at week 1 (p = 0.0477, p = 0.0113), but not for other pain scores or subsequent time points (p >0.05). CMS in TG (60.3 ±8.6) was not different from CG (59.3 ±8.8, p = 0.884) after 12 weeks or 24 weeks (71.6 ±8.1 vs. 69.9 ±7.2, p = 0.352). SST in TG (7.7 ±1.8) was not different from CG (6.9 ±2.7, p = 0.523) after 12 weeks or after 24 weeks (8.2 ±1.7 vs. 8.1 ±0.9, p = 0.292)	Tendisulfur alleviated short and partially mid-term pain after SSP tendon repair, while long-term pain was unchanged.
Gumina et al. (2012) [34]	RCT	To determine whether the intake of an oral integrator might mitigate shoulder pain and improve repair integrity of RC shoulder tear after arthroscopic repair	87 RC patients who underwent surgical repair (47-69 y, 48% male)	Two daily sachets Tenosan (arginine-L-alpha-ketoglutarate, methyl-sulfonyl-methane, hydrolyzed type I collagen and bromelain) for 3 months starting from postoperative day 1	Motion and strengthening exercises	Motion and strengthening exercises	Shoulder pain (VAS); CMS; shoulder function (SST); maximum strength; repair integrity according to Sugaya’s classification (MRI)	After 6 months: Pain decreased more in TG (−6.7) compared to CG (−5.0, p <0.001). After 12 months: no differences between groups in CMS (TG: 21.3 ±4.6, CG: 22.6 ±6.6, p = 0.329) and SST (TG: 6.9 ±1.4, CG: 7.0 ±1.9, p = 0.072). The groups were different in terms of repair type (I, II, III) (p = 0.045).	Use of the supplement for 3 months after RC repair decreases postoperative shoulder pain and leads to slight improvement in repair integrity.
Notarnicola et al. (2012) [38]	RCT	To assess the clinical efficacy and perfusion effects of oral dietary supplements in association with ESWT for insertional AT tendinopathy	64 insertional AT tendinopathy patients (55.8 ±13.2 y, 53% male)	Two daily sachets Tenosan (500 mg arginine-L-alpha-ketoglutarate, 550 mg methyl-sulfonyl-methane, 300 mg hydrolyzed collagen type I, 125 mg Vinitrox, 50 mg bromelain, 60 mg vitamin C) for 60 days	ESWT	Placebo + ESWT	Pain (VAS); subjective scores of pain and function and objective scores of physical examination (Ankle-Hindfoot Scale); pain and limitations of activity (Roles and Maudsley score)	VAS scores were lower in TG compared to CG after 2 months (3.9 ±3.2 vs. 5.1 ±2.7, p = 0.07) and 6 months (2.9 ±2.3 vs. 2.0 ±1.8, p = 0.04). Values for the Ankle-Hindfoot Scale were higher in TG compared to CG after 2 months (85 ±12.4 vs. 72.1 ±23.1, p = 0.0035) and 6 months (92.4 ±8.5 vs. 76.5 ±21.6, p = 0.0002). Roles and Maudsley scores were lower in TG compared to CG after 2 months (1.7 ±0.9 vs. 2.8 ±0.4, p <0.0001) and 6 months (1.5 ±0.6 vs. 2.3 ± 0.8, p <0.0001).	Dietary supplement plus ESWT can induce better clinical and functional outcome in AT patients.
Baar (2019) [45]	Case study	To determine whether a targeted loading and nutritional program could enhance the outcomes of a PT tendinopathy rehabilitation program	1 professional basketball player (21 y, male) with PT tendinopathy	15 g gelatine + 225 mg vitamin C twice a week for 18 months (one hour before every PT targeted training session)	Strength-based rehabilitation program	Pre-measurements	Maximal single-leg isometric hamstring strength; isometric leg extension strength; leg press strength; tendon thickness (MRI)	After 18 months: Increased hamstring (196%), leg extension (156%) and leg press (187%) strength. Thickness of proximal end of the tendon decreased by 25%. Thickness at tendon midpoint increased by 10%.	A nutritional intervention combined with a rehabilitation program can improve clinical outcomes in elite athletes
Mavrogenis et al. (2004) [37]	RCT	To evaluate the effect of essential fatty acids, antioxidants and physiotherapy on chronic tendon disorder	31 active recreational athletes with chronic tendon disorder (TG: 31 y, 76% male; CG: 32 y, 86% male)	8 capsules/day 376 mg eicosapentaenoic acid (EPA), 264 mg docosahexaenoic acid (DHA) and 672 mg gamma-linolenic acid (GLA) + 1 antioxidant-complex tablet 100 µg selenium, 15 mg zinc, 1 mg vitamin A, 2.2 mg vitamin B6, 90 mg vitamin C and 15 mg vitamin E for 32 days	Physiotherapy (therapeutic ultrasound), 16 sessions x 5 min	Placebo + physiotherapy (as TG)	Pain during sporting activity and after an isometric test (VAS); quantification of sports activity	After 32 days: Pain during sporting activity and after an isometric test decreased more in TG (99%, 99%) compared to CG (31%, 37%, p <0.001). Sports activity increased by 53% in TG and 11% in CG.	Essential fatty acids and antioxidants in combination with physiotherapy have beneficial effects in treating chronic tendon disorders.
Sandford et al. (2018) [41]	RCT	To compare the effectiveness of long chain omega-3 polyunsaturated fatty acids (PUFAs) as part of the management for people diagnosed with RC-related shoulder pain	73 patients with RC-related shoulder pain (TG: 52.2 ±12.0 y, 45% male; CG: 52.0 ±16.2 y, 57% male)	9 daily capsules MaxEPA (170 mg eicosapentaenoic acid, 115 mg docosahexaenoic acid, 2 units/g tocopherols acetate (vitamin E)) for 2 months	Weekly exercise and education groups for 8 weeks	Placebo (with same amount of vitamin E + antioxidants as TG) + weekly exercise and education groups for 8 weeks	Disability (OSS, SPADI); Pain (NRS, SF-36 bodily pain domain); Quality of life (SF-36, Euro QoL 5D-3 L); Function (PSFS); global perception of change; impairment measures (shoulder range of motion, strength).	Improved OSS scores of 25% in both groups, without differences between groups at 2 (−0.1, p = 0.95) and 12 months (−0.3, p = 0.82). SPADI scores differed only at 3 months between TG (25.3 ±21.1) and CG (13.9 ±18.1). Other outcomes improved in both groups without statistically significant differences between groups.	Omega-3 PUFA supplementation may have a modest effect on disability and pain outcomes in patients with RC-related shoulder pain at 3 months, but not over the course of one year.
Farup et al. (2014) [42]	Non-randomized controlled study	To investigate the effect of 12 weeks of either maximal eccentric or concentric resistance training combined with either a high-leucine whey protein hydrolyzate + carbohydrate supplement or placebo, on quadriceps muscle and PT hypertrophy	22 healthy young recreationally active men (23.9 ±0.8 y)	A drink containing 19.5 g high-leucine (14.2%) whey protein hydrolyzate + 19.5 g carbohydrate (glucose) on all training days (33x in 12 weeks)	Eccentric training with one leg, concentric training with the other leg	Placebo (isoenergetic carbohydrate (glucose)) + training (as TG)	PT CSA (MRI); isometric strength (MVC, RFD)	After 12 weeks: Greater increase in PT CSA at proximal level in TG (14.9 ±3.1%) compared to CG (8.1 ±3.2%, p = 0.054). MVC and RFD increased by 15.6 ±3.5% (p <0.001) and 12-63% (p <0.05) without group effects.	Training-induced hypertrophy of the PT was augmented with a high-leucine whey protein hydrolyzate supplement.
Saggini et al. (2010) [40]	RCT (two-arm)	To evaluate the efficacy of a specific rehabilitative, therapeutic protocol integrated with administration of a supplement in both conservative rehabilitation treatment and post-surgery, in patients with RC lesions	Arm A: 30 RC lesion patients, treated conservatively (45 ±10 y, 37% male). Arm B: 50 RC lesion patients, treated conservatively (59.5 (30-80) y (female), 58.4 (28-78) y (male), 48% male)	1 sachet/day 3.5 g Amedial BF (glucosamine sulfate, chondroitin sulfate, hydrolyzed type II collagen, hydrolyzed hyaluronic acid, L-carnitine fumarate) for 1 month (Arm A) for 60 days (Arm B)	Arm A: 3 shock waves + 9 sittings Multi Joint System; Arm B: rehabilitation treatment	Arm A: 3 shock waves + 9 sittings Multi Joint System (CG1) or 3 shock waves (CG2); Arm B: rehabilitation treatment	Arm A: ROM; pain (VAS); Arm B: UCLA (pain, functionality, active frontal flexion, strength in frontal flexion, satisfaction)	Arm A, after 1 month: VAS scores reduced by 45% in TG, 22% in CG1 and 45% in CG2. Flexion, extension, abduction and external rotation increased by 38%, 57%, 47% and 52% in TG, 28%, 40%, 42% and 40% in CG1 and 26%, 23%, 36% and 25% in CG2. Arm B, after 60 days: Higher improvement in TG compared to CG for pain (73% vs. 70%), function (49% vs. 36%), flexion (41% vs. 29%) and strength (39% vs. 30%) scores. Satisfaction was higher in TG (92%) than in CG (84%).	Supplementation of natural substances is a conservative treatment for RC lesions to consider. Quicker functional recovery with post-surgical supplementation.
Schneider et al. (2009) [46]	Case series	To identify characteristics associated with bilateral ruptures of the distal biceps tendons	10 patients with sustained non-simultaneous bilateral distal biceps brachii tendon ruptures, surgically repaired (49.5 (27.7-76.2) y, male)	Nutritional supplements (multivitamins and omega 3 oils)	NA	NA	Disability (DASH)	DASH scores were not significantly related to using nutritional supplements at the time of injury (p = .145)	No correlation found between outcome following surgical treatment and use of nutritional supplements.
Szczurko et al. (2009) [28]	RCT	To evaluate the potential for the combined efficacy of a naturopathic approach including acupuncture, dietary advice and hydrolytic enzymes in the treatment of RC tendinitis	85 Canadian postal employees with RC tendinitis (TG: 50.7 ±8.16 y, 42% male; CG: 50.9 ±7.86 y, 40% male)	6 tablets/day of Phlogenzym (90 mg bromelain, 48 mg trypsin, 100 mg rutin) + patient-customized dietary counseling, with special emphasis on reducing alcohol consumption and increasing consumption of fish, berries, fruits, vegetables, nuts, and whole grains for 12 weeks	Acupuncture	Placebo + physical exercise	Disability (SPADI); health-related QoL (SF-36); pain over the last week (VAS); patient experiences (MYMOP); flexion, extension, abduction, adduction, internal rotation and external rotation of affected shoulder	After 12 weeks: SPADI scores improved more in TG (54.5%) compared to CG (18%, p <0.0001). TG also showed superiority in SF-36, VAS, MYMOP scores and range of motion.	Naturopathic treatments including dietary changes, acupuncture and Phlogenzym have a significant effect on decreasing RC tendinitis symptoms.

AT, Achilles tendon; CG, control group; CMS, Constant-Murley score; CSA, cross-sectional area; DASH, Disabilities of Arm, Shoulder, and Hand; ESWT, Extracorporeal Shock Wave Therapy; LET, lateral epicondyle tendon; MEPS, Mayo Elbow Performance Score; MMYOP, Measure Yourself Medical Outcomes Profile; MRI, magnetic resonance imaging; MVC, maximal voluntary contraction; NRS, Numerical Rating Scale; OSS, Oxford Shoulder Score; PRTEE, Patient-rated Tennis Elbow Evaluation; PSFS, Patient-Specific Functional Scale; PT, patellar tendon; RC, rotator cuff; RCT, randomized controlled trial; RFD, rate of force development; SF-36, Short-Form Health Survey; SPADI, Shoulder Pain and Disability Index; SSP, supraspinatus; SST, Simple Shoulder Test; TG, treatment group; RC, rotator cuff; VAS, Visual Analogue Scale; VISA-A, Victorian Institute of Sports Assessment – Achilles questionnaire; VISA-P, Victorian Institute of Sports Assessment – Patellar questionnaire; USI, ultrasound imaging.

### Study designs and populations

All studies examining habitual dietary intake had an observational design; there were two prospective cohort studies [29,30], two cross-sectional studies [31,32] and one case-control study [33]. The number of people included in these studies ranged from 50 to 80,106 (Table 1).

Among the fourteen studies examining the use of dietary supplements, thirteen were experimentally designed: nine RCTs [28,34–41], two non-randomized controlled studies [42,43], one non-comparative intervention study [44] and one case study [45]. In addition, there was one retrospective case series [46]. The number of subjects in these studies ranged from 1 to 100 (Table 2).

Although there was a wide age range within the study populations, they mainly comprised middle-aged (40-60 years) and older (>60 years) non-athletic adults. The participants in three studies examining athletes or active persons were younger [37,42,45]. Also, one study among military personnel had a large proportion of young adults [30]. Most studies included both men and women, but some included relatively more [29,37] or only men [31,42,45,46].

### Location of tendon/tendinopathy

Tendons of the rotator cuff were investigated in nine studies [28,29,32–35,40,41,43]. The Achilles tendon was examined in seven studies [30,31,36,38,39,43,44], the patellar tendon in four [30,42,44,45], the lateral elbow tendon in two [43,44] and the biceps brachii tendon in one study [46]. One study did not specify the tendons of interest, but included several types [37]. The effect of nutritional exposure to healthy tendons was investigated in two studies [31,42]. A total of seventeen studies examined people with prevalent tendinopathy who are at risk of tendinopathy [28–30,32–41,43–46].

### Nutritional exposure

In the studies investigating the impact of the habitual diet, questionnaires were used to assess nutritional exposure. One study examined the intake of several foods as well as the overall dietary pattern (Western vs Mediterranean) [31], and four studies examined exposure to alcohol [29,30,32,33]. The majority of studies examined the effect of a dietary supplement that contained multiple ingredients, including collagen [34–36,38–40,43,44], vitamin C [36–38,43–45], methyl-sulfonyl-methane [34,35,38,43], arginine-L-alpha-ketoglutarate [34,38], mucopolysaccharides [36,44], bromelain [28,34,38] and essential fatty acids [37,41]. One study evaluated an intervention combining supplements and habitual dietary changes [28]. Duration of supplement use ranged from 1 to 18 months.

### Concurrent interventions

Twelve out of the thirteen experimental studies implemented other interventions in addition to the use of dietary supplements. The concurrent exposures were training or exercises [34,36,39,41,42,45], analgesic therapy [35], Extracorporeal Shock Wave Therapy (ESWT) [38,40,43], physiotherapy [37], multi-joint system [40], rehabilitation treatment [40] and acupuncture [28]. In addition, six studies considered patients that underwent surgical repair of either the rotator cuff tendon [29,33–35,40] or the biceps brachii tendon [46].

### Outcome measures

Clinical outcomes were reported in fifteen studies. Pain was the clinical outcome reported most often, and was measured by the visual analogue scale (VAS) [28,34–38,40,43,44], numerical rating scale (NRS) [41] or Short-Form 36 bodily pain (SF-36 BP) [41]. Disability was captured by many different outcomes, including the Shoulder Pain and Disability index (SPADI) [28,29,41], Victorian Institute of Sport Assessment for the Achilles (VISA-A) [36,39,43,44] or patellar tendon (VISA-P) [44], Patient-rated Tennis Elbow Evaluation (PRTEE) [44], Roles and Maudsley score [38], Oxford Shoulder Score (OSS) [41] and Disabilities of Arm, Shoulder and Hand (DASH) score [46]. Physical function capacity was examined by measuring strength [34,41,42,45], range of motion [28,40,41] and function with the simple shoulder test (SST) [34,35] and Patient-Specific Functional Score (PSFS) [41]. Combinations of clinical outcomes were assessed by the UCLA shoulder score [40,43], Mayo Elbow Performance Score (MEPS) [43], Ankle-Hindfoot Scale [38] and Constant-Murley score [34,35]. Other outcomes were global perception of change [41] and Measure Yourself Medical Outcomes Profile (MYMOP) [28] to measure patient rating of condition, SF-36 [28,41] and Euro QoL 5D-3 [41] to measure quality of life, and quantification of sports activity [37] (participation in life activities). None of the studies assessed psychological factors.

Three studies calculated odds ratios as a measure of association between exposure to alcohol and occurrence/prevalence of tendinopathy [30,32,33]. Structural changes of the tendon by MRI or USI were examined in seven studies [31,34,36,39,42,44,45].

### Risk of bias

The risk of bias assessment of all nineteen eligible studies, including the most important sources of bias, is presented in Tables 3 and 4. For the nine RCTs this was evaluated with the RoB 2 tool (Table 3). Two studies were judged at low risk of bias [38,41]; both were double-blinded, placebo-controlled, and included several outcome measures for which the results were reported adequately. However, one of these studies [38] raised some small concerns because baseline characteristics were not presented. Six studies expressed ‘some concerns’ [28,34–37,39]. Main aspects that raised concerns were no inclusion of placebo treatment [34,36], small study populations [36,37,39], unclear or imbalance of baseline comparison and/or other confounding factors [36,39], and mainly presenting participant-reported outcomes [35,37]. Additional concerns, involving judgment tending toward high risk of bias, were raised by the absence of intention-to-treat analyses, assessment of limited outcomes and short follow-up [37], and a cross-over design without a wash-out period [39]. One study had a high risk of bias due to poor clarity of the randomization process, participant characteristics, study protocol and analyses, and selective reporting of results [40].

**Table 3.** Overall quality judgment of each study assessed by the RoB 2 tool.

Author	Overall Risk of Bias Judgment*	Main Sources of Bias
Balius et al. [36]	Some concerns	No placebo treatment Small study population No baseline comparison
Gumina et al. [34]	Some concerns (high)	No placebo treatment
Mavrogenis et al. [37]	Some concerns (high)	No intention-to-treat analyses Small study population with broad inclusion criteria Only participant-reported outcomes Short follow-up
Merolla et al. [35]	Some concerns	Mainly participant-reported outcomes No between-group comparison of change
Notarnicola et al. [38]	Low risk (some concerns)	No baseline characteristics presented
Praet et al. [39]	Some concerns (high)	No wash-out period Small study population Potential of selection bias
Saggini et al. [40]	High risk	Insufficient information about randomization, group comparison, protocol and analyses Incomplete and unclear reporting of results
Sandford et al. [41]	Low risk	
Szczurko et al. [28]	Some concerns	High drop-out rate Individual variability in the multiple components of the intervention delivered

*Possible judgments are: Low risk, Some concerns, High risk

**Table 4.** Overall quality judgment of each study assessed by the ROBINS-I tool.

Author	Overall Risk of Bias Judgment*	Main Sources of Bias
Arquer et al. [44]	Serious	No control group High drop-out rate Limited participant information
Baar [45]	Serious	One participant
Farup et al. [42]	Low	Small study population
Hjerrild et al. [31]	Serious	Cross-sectional design Incomprehensive exposure assessment Not all confounders were taken into account
Jain, et al. [29]	Moderate/Serious	Small study population Participant-reported outcome Incomplete follow-up
Owens et al. [30]	Moderate	Not all confounders were taken into account Only severe cases were identified Inappropriate statistical adjustments
Passaretti et al. [33]	Serious	Potential of selection bias Potential for underreporting of alcohol consumption
Rechardt et al. [32]	Moderate	Cross-sectional design Data of association not shown
Schneider et al. [46]	Critical	Small study population Selection bias No quantification and qualification of exposure Single participant-reported outcome measure
Vitali et al [43].	Moderate	No placebo Mainly participant-reported outcomes

*Possible judgments are: Low, Moderate, Serious, Critical

The ROBINS-I tool was used to assess the risk of bias of the ten remaining studies (Table 4). One study was judged at low risk of bias, although the study population was small [42]. Other studies presented limitations as a result of their observational study design [29–33], small study populations [29,45], incomplete follow-up [29,44] and inappropriate exposure or outcome assessment [29–31,33,43], and were therefore judged at moderate [29,30,32,43] or serious [31,33,44,45] risk of bias. The case series [46] was judged as critical because of selection bias in its small study population, insufficient information about exposure, and assessment of one single outcome measure.

The overall level of certainty in the evidence for clinical outcomes was based on fourteen studies that examined the effect of a dietary supplement (Additional file 5). For the occurrence/prevalence of tendinopathy, the overall assessment of certainty included three studies that examined the effect of alcohol intake (Additional file 6). The summary of findings regarding the judgments of the certainty in evidence is presented in Additional file 7. For the clinical outcomes, the certainty in the evidence was judged at low. There was a very low level of certainty for occurrence/prevalence of tendinopathy.

## 4. Discussion

This systematic review aimed to evaluate the potential impact of nutrition on the prevention and treatment of tendinopathy. The majority of the included studies investigated the use of dietary supplements. Only a limited number of studies examining the effect of the habitual diet on tendon outcomes were identified. Overall, there was insufficient high-quality data available to enable meta-analyses as a result of the considerable variation in study design, nutritional exposure, concurrent exposure, outcome measures, and risk of bias.

### Habitual diet

Only one study was identified that investigated the habitual intake of several foods and type of diet [31]. This study did not find an association between any of the dietary parameters and Achilles tendon thickness, but no other clinical outcomes were assessed. Considering the serious risk of bias, firm conclusions about the impact of the habitual diet from this single study are not possible.

### Alcohol

Findings of the four studies examining the impact of alcohol intake varied for effect on tendinopathy. No associations were found between alcohol consumption and chronic rotator cuff tendinitis [32]. Moderate weekly alcohol consumption (men: 7-13 drinks, women: 4-6 drinks) was associated with a modest increased risk for Achilles tendinopathy, but not with patellar tendinopathy [30]. Excessive alcohol intake (men: >13 drinks, women: >6 drinks) was a significant risk factor for the occurrence and severity of rotator cuff tears [33]. By contrast, Jain et al. [29] found a positive association between alcohol consumption and less shoulder pain and better function after rotator cuff repair. However, it was suggested that alcohol use may be a proxy for another variable that was not captured in the study. In addition, differences in study design, population, and classification into categories for amount of alcohol consumption (light, moderate, heavy) limit comparability across these studies. Despite inconsistent findings on the risk and severity of tendinopathy in humans, there is evidence that alcohol may inhibit collagen synthesis through toxic effects [47].

### Collagen

As tendons are mainly composed of collagen, whose ongoing synthesis is required to maintain a healthy extracellular matrix, it is not surprising that collagen-derived peptides, including gelatin and hydrolyzed collagen, were most often a component of the dietary supplements evaluated. The majority of these supplements were found to improve clinical and/or structural outcomes in the treatment of tendinopathy [34,36,38–40,43–45]. Merolla et al. [35] showed only short-term effectiveness for pain reduction after supraspinatus tendon repair, while long-term pain was unchanged. This could be addressed by increased dosage and treatment duration. Thus, in the study of Gumina et al. [34], an extended treatment of a similar preparation in rotator cuff patients showed improvement in pain after six months. However, both studies did not find a better physical function capacity after surgical repair as a result of the supplement intake. In all other studies examining the effect of a supplement containing collagen, participants were not treated surgically and benefits for various clinical outcomes were found [36,38–40,43–45]. Although most studies showed improvement after 2-3 months of supplement use, the daily dose as well as the type of collagen varied among interventions. Tendon is mainly composed of type I, while cartilage contains type II. Most tendon studies supplemented type I, although some combined with type II [35,43], or type II only [40], which can explain some of the variation in outcome. All in all, collagen seems to be beneficial in the treatment of tendinopathy, but conclusions about optimal dosage, timing, duration, and type of collagen supplementation cannot be drawn yet. Also, because many interventions implemented collagen supplementation with concurrent treatment, the specific benefits of collagen alone remain unknown.

### Other nutrients

Many other nutrients may contribute to collagen synthesis or may have anti-inflammatory effects, so most dietary supplements used in the different studies contained multiple substances. In four studies a similar preparation was used, as they all comprised methyl-sulfonyl-methane and arginine in addition to hydrolyzed collagen [34,35,38,43]. Curcumin and Boswellia serrata were only used by Merolla et al. [35] and Vitali et al. [43], while the preparations used by Gumina et al. [34] and Notarnicola et al. [38] contained bromelain. All these compounds were found to be associated with improvement in pain, but a specific evaluation of each nutrient is difficult because of the multiple nutrients within a single supplement.

Also, the amino acid leucine may have exerted a stimulatory effect on collagen synthesis. A single study in healthy young men found that tendon hypertrophy was augmented with high-leucine whey protein supplementation (providing 19.5 g amino acids of which 2.77 g leucine on training days) in addition to resistance exercise [42]. Although findings from this small single study are not conclusive, they may have important clinical implications. Leucine-induced tendon hypertrophy may lead to relatively less mechanical stress on the tendon during exercise, which may assist in tendon rehabilitation.

Two studies investigated the potential role of essential fatty acids in the treatment of tendinopathy because of their anti-inflammatory properties [48]. In the high-quality trial of Sandford et al. [41], eight weeks of Omega 3 PUFA supplementation (daily providing 1530 mg of EPA and 1035 mg of DHA) was found to have a modest improvement on disability and pain outcomes in patients with rotator cuff-related shoulder pain after 3 months, but not after one year. Mavrogenis et al. [37] used supplements for only 32 days but at a higher dose, and reported a significant improvement in pain. However, the short follow-up and other methodological concerns limit the potential impact of these findings. Current evidence does not support the use of essential fatty acids, but further research is warranted to assess the potential impact of higher-dose and longer-duration interventions.

The intervention evaluated by Szczurko et al. [28] consisted of multiple components, including supplementation of hydrolytic enzymes (bromelain, trypsin, rutin), individual dietary counseling, and acupuncture. This naturopathic treatment showed clinically significant improvement in shoulder pain and quality of life compared with standardized physical exercise. Although they raised only small concerns with respect to risk of bias, their study is of limited evidence for the effectiveness of dietary supplements and/or dietary changes on tendinopathy, because the effects of the individual components cannot be established.

### Study quality and limitations

Despite the broad inclusion criteria, the total number of eligible studies was relatively low. In addition, the overall quality of studies was poor. Evidence for the effects of nutrition results from limited high-quality studies. An overall rating of the certainty in the evidence was only provided for clinical outcomes and for occurrence/prevalence of tendinopathy.

Another limitation resulting from the inclusion criteria is the heterogeneity among studies. This is why the rating of quality of evidence needs to be interpreted with caution. Especially the evidence from studies on the effects of nutrition in healthy human tendons was scarce. We also included studies that did not primarily aim to investigate nutritional exposure or one of the eligible outcomes, and therefore did not assess or report this in much detail. Nevertheless, this review provides an overview of the evidence for effects of any nutritional exposure and is the first study to systematically synthesize the findings for clinical outcomes on physical, psychosocial and overall life impact, and the risk of tendinopathies in relation to nutrition.

A limitation of the included studies is that supplement use was often combined with several types of interventions (e.g. exercises, physiotherapy, shockwaves, surgery). Although these interventions were similar in the control group, it cannot be ruled out that the concurrent treatment supported the effect of the nutritional treatment. The results show that combining supplement use with other treatments provides further benefits than the treatment alone. It would be interesting to see what effects are induced by taking the dietary supplement alone. Nevertheless, nutritional strategies do not interfere and can easily be implemented in combination with other interventions.

Another barrier in the synthesis of findings from the different studies is that many disparate clinical outcomes were reported. Many studies rely on a limited number of outcomes and were mainly participant-reported. Pain measured by the VAS was an outcome reported in most studies, but referred to a different activity or timeframe, or rated pain without further specification. As recommended by the ICON group, clinical trials should include a measure for each of the nine core domains at a minimum [23]. However, from the studies identified in this review that of Sandford et al. [41] captured six domains. Other studies reported even less. This stresses the need to determine a core outcome set that should be adopted widely in tendinopathy research.

### Recommendations for future research

More knowledge is required on the impact of habitual dietary exposures on tendon health, as a healthy habitual diet is the basis for adequate nutrient intake. Improving the habitual intake should be the main focus of athletes rather than the intake of dietary supplements. High-quality studies with extensive dietary intake assessment are needed to examine this association in tendinopathy patients as well as in healthy populations, to determine the role of nutrition in preventing tendinopathy. Research should be conducted specifically in athletes and active populations and by assessing core clinical outcome measures to enable future meta-analyses.

## 5. Conclusion

Due to the limited scientific quality and variety among studies on nutrient intake, tendon location, study population and reported outcome measures, it is impossible to draw definitive conclusions and formulate dietary recommendations on the prevention and treatment of tendinopathy. Findings on alcohol intake were inconsistent. Individual studies present important clinical implications for the use of dietary supplements on tendon health, of which especially those containing collagen-derived peptides seem to be beneficial in the treatment of tendinopathy. Also, methyl-sulfonyl-methane, arginine, bromelain, curcumin, and Boswellia were present in supplements that showed clinical improvements. Future clinical studies considering nutritional intake should use standardized dietary assessment methods, adopt the core domains for tendon research and report a core outcome set for each tendinopathy, in order to synthesize findings from different studies.

## Supplementary Material

Supplemental Material

Supplemental Material

Supplemental Material

Supplemental Material

Supplemental Material

## Data Availability

The datasets generated during the current study are available from the corresponding author on reasonable request.

## Additional file 1

**Table:** **Table 1** Core domains of tendinopathy as defined by the ICON group [2019, 23].

Domain	Description/definition	Example outcome
Patient rating of condition	A single assessment numerical evaluation	Rate your tendon status where 100% is no problems and 0% worst case scenario, global rating of change, patient acceptable symptom status
Participating in life activities	Patient rating of the level of participating	Ratings of level of sport and time to return to sport
Pain on activity/loading	Patient reported intensity of pain on performing a task/activity that loads the tendon	VAS or NRS for pain intensity when the patient performs a tendon-specific pain-provocative task
Function	Patient rated level of function (and not referring to the intensity of their pain)	Patient Specific Function Scale on a VAS or NRS
Psychological factors	Psychology	Pain self-efficacy, pain catastrophisation, kinesiophobia, anxiety or depression scales
Physical function capacity	Quantitative measures of physical tasks performed in clinic	Number of hops, timed stair walk, number of single limb squats, including dynamometry and wearable technology
Disability	Composite scores of a mix of patient-rated pain and disability due to the pain, usually to tendon-specific activities/tasks	VISA scales, patient-rated tennis elbow evaluation, disability of the arm, shoulder and hand
Quality of life	The general well-being of the individual	Specific QoL questionnaires such as European QoL – 5 Dimension (EQ-5D) Australian QoL (AQoL), 36-item Short Form survey (SF-36)
Pain over a specified time	Participant reported pain intensity over a period of time (morning, night, 24 hours, a week)	VAS, NRS

## Additional file 2

**Table:** **Table 2** Search strategy in PubMed.

Concept	Search terms
Tendinopathy	(((‘Tendinopathy’[Mesh] OR tendinopathy[tiab] OR tendinopathies[tiab] OR tendinosis[tiab] OR tendinoses[tiab] OR tendinitis[tiab] OR tendonitis[tiab] OR tendonosis[tiab] OR tendonitides[tiab] OR ‘Tendon Injuries’[Mesh] OR tendon injuries[tiab] OR tendon injury[tiab] OR tendon healing[tiab] OR tendon disorder*[tiab] OR tendon repair[tiab]) OR ((‘Tendons’[Mesh] OR tendon*[tiab]) AND (‘prevention and control’ [Subheading] OR prevention[tiab] OR preventive therapy[tiab])))
Nutrition	AND (curcumin[tiab] OR boswellic acid[tiab] OR arginin*[tiab] OR tendisulfur[tiab] OR bromelain[tiab] OR methylsulfonylmethane[tiab] OR ‘Amino Acids, Peptides, and Proteins’[Mesh] OR amino acid[tiab] OR protein[tiab] OR proteins[tiab] OR leucine[tiab] OR glutamine[tiab] OR arginine[tiab] OR taurine[tiab] OR gelatin[tiab] OR ‘Collagen’[Mesh] OR collagen[tiab] OR ‘Phytochemicals’[Mesh] OR phytochemicals[tiab] OR phytonutrients[tiab] OR ‘coenzyme Q10’[Supplementary Concept] OR coenzyme Q10[tiab] OR co-enzyme Q10[tiab] OR ‘Fatty Acids, Omega-3’[Mesh] OR omega 3[tiab] OR omega-3[tiab] OR ‘Lipids’[Mesh] OR lipids[tiab] OR fatty acids[tiab] OR fish oils[tiab] OR plant oils[tiab] OR ‘Nutrition Therapy’[Mesh] OR nutrition therapy[tiab] OR diet therapy[tiab] OR nutrient intake[tiab] OR ‘Nutrients’[Mesh] OR nutrient*[tiab] OR macronutrient*[tiab] OR ‘Diet, Food, and Nutrition’[Mesh] OR nutrition[tiab] OR ‘Micronutrients’[Mesh] OR micronutrient*[tiab] OR vitamin*[tiab] OR ‘Ascorbic Acid’[Mesh] OR ascorbic acid[tiab] OR vitamin c[tiab] OR antioxidant*[tiab] OR ‘Vitamin D’[Mesh] OR vitamin d[tiab] OR cholecalciferol[tiab] OR ergocalciferols[tiab] OR ‘Minerals’[Mesh] OR minerals[tiab] OR calcium[tiab] OR manganese[tiab] OR copper[tiab] OR zinc[tiab] OR magnesium[tiab] OR iron[tiab] OR molybdenum[tiab] OR silicon[tiab] OR calories[tiab] OR ‘Dietary Supplements’[Mesh] OR dietary supplement*[tiab] OR food supplement*[tiab] OR food additives[tiab] OR fortified food[tiab] OR nutraceutical[tiab] OR nutritional[tiab] OR ‘Glycerol’[Mesh] OR glycerin[tiab] OR glycerol[tiab]))
Human	NOT ((animals[mh] NOT (animals[mh] AND humans[mh])) NOT rat[tiab] NOT rats[tiab] NOT mice[tiab] NOT rabbit*[tiab])

## Additional file 3

**Table:** **Table 3** Rating of the certainty of evidence for clinical outcomes.

GRADE domain	Judgment	Concerns about certainty domains
Methodological limitations of the studies	Among the nine RCTs, the majority expressed ‘some concerns’ with respect to the risk of bias. Two studies were judged at low risk of bias and one study had a high risk of bias. The risk of bias of the remaining five intervention and observational studies was judged at low or moderate for two studies, and serious or critical for three studies. Main aspects that raise concerns were reporting participant-reported outcomes, incomplete or unclear reporting of methods or results, and small study populations. Therefore, we judged the studies to have serious methodological limitations.	Serious
Indirectness	Most studies were primarily aimed to investigate the effect of the dietary supplement on clinical outcomes, but often in combination with other treatments. We judged the evidence to have moderate indirectness.	Moderate
Imprecision	The total number of participants included in all studies was 819. We judged the evidence to have moderate imprecision.	Moderate
Inconsistency	The majority of the studies found a beneficial effect of the supplement intake on one or more of the clinical outcomes. There is inconsistency in the effects on different time points, but this could be addressed by variation in study protocol. We judged the evidence to have moderate inconsistency.	Moderate
Publication bias	Some studies are commercial studies. We found no commercial studies without effect.	Potential

## Additional file 4

**Table:** **Table 4** Rating of the certainty of evidence for occurrence/prevalence of tendinopathy.

GRADE Domain	Judgment	Concerns About Certainty Domains
Methodological limitations of the studies	The risk of bias was judged at moderate for two out of three studies. One study was judged at serious risk of bias, but this study was smaller compared to the other two (605 vs. 80,106/6237). All studies had an observational design, which involves several limitations. In addition, sources of bias were inappropriate statistical adjustments, potential underreporting of intake and incomplete reporting of results. Therefore, we judged the studies to have serious methodological limitations.	Serious
Indirectness	Only one study primarily aimed to investigate the association between the intake of alcohol and the risk of tendinopathy. In the other two studies, alcohol consumption was only one of many factors that were investigated to find an association. One study did not even report data with regard to alcohol consumption. Therefore, we judged the evidence to have serious indirectness.	Serious
Imprecision	The total number of participants included in all studies was 86,948. This is a large number, but this is mainly due to one large cohort study with relatively low number of cases identified. We judged the evidence to have moderate imprecision.	Moderate
Inconsistency	The studies reported either a positive association or no association between alcohol consumption and the risk of tendinopathy. One study found a marginal association for moderate weekly alcohol consumption and Achilles tendinopathy, but not for heavy weekly alcohol consumption or patellar tendinopathy. Another study found significant risks of rotator cuff tears for excessive drinkers. We judged the evidence to have moderate inconsistency.	Moderate
Publication bias	We do not suspect publication bias, taking into account that we have few studies	Not suspected

## Additional file 5

**Table:** **Table 5** Summary of findings regarding the GRADE judgments.

Outcome	Effect	Number of Participants (Studies)	Certainty in the Evidence
Clinical outcomes	Most studies showed positive effects on one or more clinical outcomes, or found no significant effects	819 (14 experimental studies including 9 RCTs)	Low
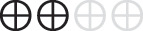
Occurrence/prevalence of tendinopathy	Two studies found a positive association between alcohol consumption and risk of tendinopathy. One study showed no association.	86,948 (3 observational studies)	Very low
