# Supplementary material for: The impact of nutrition on tendon health and tendinopathy: a systematic review
Source: J Int Soc Sports Nutr. 2022 Aug 3;19(1):474–504. doi: 10.1080/15502783.2022.2104130 (PMC9354648; doi:10.1080/15502783.2022.2104130)
Supplement: Supplemental Material [file RSSN_A_2104130_SM1029.docx]

**Additional file 5**

**Table 5** Summary of findings regarding the GRADE judgements

| Outcome | Effect | Number of participants (studies) | Certainty in the evidence |
| --- | --- | --- | --- |
| Clinical outcomes | Most studies showed positive effects on one or more clinical outcomes, or found no significant effects | 819 (14 experimental studies including 9 RCTs) | ⊕⊕⊕⊕  Low |
| Occurrence/prevalence of tendinopathy | Two studies found a positive association between alcohol consumption and risk of tendinopathy. One study showed no association. | 86,948 (3 observational studies) | ⊕⊕⊕⊕  Very low |
