# Supplementary material for: The impact of nutrition on tendon health and tendinopathy: a systematic review
Source: J Int Soc Sports Nutr. 2022 Aug 3;19(1):474–504. doi: 10.1080/15502783.2022.2104130 (PMC9354648; doi:10.1080/15502783.2022.2104130)
Supplement: Supplemental Material [file RSSN_A_2104130_SM1028.docx]

**Additional file 4**

**Table 4** Rating of the certainty of evidence for occurrence/prevalence of tendinopathy

| GRADE domain | Judgement | Concerns about certainty domains |
| --- | --- | --- |
| Methodological limitations of the studies | The risk of bias was judged at moderate for two out of three studies. One study was judged at serious risk of bias, but this study was smaller compared to the other two (605 vs. 80,106/6237). All studies had an observational design, which involves several limitations. In addition, sources of bias were inappropriate statistical adjustments, potential underreporting of intake and incomplete reporting of results. Therefore, we judged the studies to have serious methodological limitations. | Serious |
| Indirectness | Only one study primarily aimed to investigate the association between the intake of alcohol and the risk of tendinopathy. In the other two studies, alcohol consumption was only one of many factors that were investigated to find an association. One study did not even report data with regard to alcohol consumption. Therefore, we judged the evidence to have serious indirectness. | Serious |
| Imprecision | The total number of participants included in all studies was 86,948. This is a large number, but this is mainly due to one large cohort study with relatively low number of cases identified. We judged the evidence to have moderate imprecision. | Moderate |
| Inconsistency | The studies reported either a positive association or no association between alcohol consumption and the risk of tendinopathy. One study found a marginal association for moderate weekly alcohol consumption and Achilles tendinopathy, but not for heavy weekly alcohol consumption or patellar tendinopathy. Another study found significant risks of rotator cuff tears for excessive drinkers. We judged the evidence to have moderate inconsistency. | Moderate |
| Publication bias | We do not suspect publication bias, taking into account that we have few studies | Not suspected |
