# Supplementary material for: The impact of nutrition on tendon health and tendinopathy: a systematic review
Source: J Int Soc Sports Nutr. 2022 Aug 3;19(1):474–504. doi: 10.1080/15502783.2022.2104130 (PMC9354648; doi:10.1080/15502783.2022.2104130)
Supplement: Supplemental Material [file RSSN_A_2104130_SM1025.docx]

**Additional file 3**

**Table 3** Rating of the certainty of evidence for clinical outcomes

| GRADE domain | Judgement | Concerns about certainty domains |
| --- | --- | --- |
| Methodological limitations of the studies | Among the nine RCTs, the majority expressed ‘some concerns’ with respect to the risk of bias. Two studies were judged at low risk of bias and one study had a high risk of bias. The risk of bias of the remaining five intervention and observational studies was judged at low or moderate for two studies, and serious or critical for three studies. Main aspects that raise concerns were reporting participant-reported outcomes, incomplete or unclear reporting of methods or results, and small study populations. Therefore, we judged the studies to have serious methodological limitations. | Serious |
| Indirectness | Most studies were primarily aimed to investigate the effect of the dietary supplement on clinical outcomes, but often in combination with other treatments. We judged the evidence to have moderate indirectness. | Moderate |
| Imprecision | The total number of participants included in all studies was 819. We judged the evidence to have moderate imprecision. | Moderate |
| Inconsistency | The majority of the studies found a beneficial effect of the supplement intake on one or more of the clinical outcomes. There is inconsistency in the effects on different time points, but this could be addressed by variation in study protocol. We judged the evidence to have moderate inconsistency. | Moderate |
| Publication bias | Some studies are commercial studies. We found no commercial studies without effect. | Potential |
