# Supplementary material for: The impact of nutrition on tendon health and tendinopathy: a systematic review
Source: J Int Soc Sports Nutr. 2022 Aug 3;19(1):474–504. doi: 10.1080/15502783.2022.2104130 (PMC9354648; doi:10.1080/15502783.2022.2104130)
Supplement: Supplemental Material [file RSSN_A_2104130_SM1020.docx]

**Additional file 2**

**Table 2** Search strategy in PubMed

| Concept | Search terms |
| --- | --- |
| Tendinopathy | ((("Tendinopathy"[Mesh] OR tendinopathy[tiab] OR tendinopathies[tiab] OR tendinosis[tiab] OR tendinoses[tiab] OR tendinitis[tiab] OR tendonitis[tiab] OR tendonosis[tiab] OR tendonitides[tiab] OR "Tendon Injuries"[Mesh] OR tendon injuries[tiab] OR tendon injury[tiab] OR tendon healing[tiab] OR tendon disorder*[tiab] OR tendon repair[tiab]) OR (("Tendons"[Mesh] OR tendon*[tiab]) AND ("prevention and control" [Subheading] OR prevention[tiab] OR preventive therapy[tiab]))) |
| Nutrition | AND (curcumin[tiab] OR boswellic acid[tiab] OR arginin*[tiab] OR tendisulfur[tiab] OR bromelain[tiab] OR methylsulfonylmethane[tiab] OR "Amino Acids, Peptides, and Proteins"[Mesh] OR amino acid[tiab] OR protein[tiab] OR proteins[tiab] OR leucine[tiab] OR glutamine[tiab] OR arginine[tiab] OR taurine[tiab] OR gelatin[tiab] OR "Collagen"[Mesh] OR collagen[tiab] OR "Phytochemicals"[Mesh] OR phytochemicals[tiab] OR phytonutrients[tiab] OR "coenzyme Q10"[Supplementary Concept] OR coenzyme Q10[tiab] OR co-enzyme Q10[tiab] OR "Fatty Acids, Omega-3"[Mesh] OR omega 3[tiab] OR omega-3[tiab] OR "Lipids"[Mesh] OR lipids[tiab] OR fatty acids[tiab] OR fish oils[tiab] OR plant oils[tiab] OR "Nutrition Therapy"[Mesh] OR nutrition therapy[tiab] OR diet therapy[tiab] OR nutrient intake[tiab] OR "Nutrients"[Mesh] OR nutrient*[tiab] OR macronutrient*[tiab] OR "Diet, Food, and Nutrition"[Mesh] OR nutrition[tiab] OR "Micronutrients"[Mesh] OR micronutrient*[tiab] OR vitamin*[tiab] OR "Ascorbic Acid"[Mesh] OR ascorbic acid[tiab] OR vitamin c[tiab] OR antioxidant*[tiab] OR "Vitamin D"[Mesh] OR vitamin d[tiab] OR cholecalciferol[tiab] OR ergocalciferols[tiab] OR "Minerals"[Mesh] OR minerals[tiab] OR calcium[tiab] OR manganese[tiab] OR copper[tiab] OR zinc[tiab] OR magnesium[tiab] OR iron[tiab] OR molybdenum[tiab] OR silicon[tiab] OR calories[tiab] OR "Dietary Supplements"[Mesh] OR dietary supplement*[tiab] OR food supplement*[tiab] OR food additives[tiab] OR fortified food[tiab] OR nutraceutical[tiab] OR nutritional[tiab] OR "Glycerol"[Mesh] OR glycerin[tiab] OR glycerol[tiab])) |
| Human | NOT ((animals[mh] NOT (animals[mh] AND humans[mh])) NOT rat[tiab] NOT rats[tiab] NOT mice[tiab] NOT rabbit*[tiab]) |
