# Supplementary material for: The impact of nutrition on tendon health and tendinopathy: a systematic review
Source: J Int Soc Sports Nutr. 2022 Aug 3;19(1):474–504. doi: 10.1080/15502783.2022.2104130 (PMC9354648; doi:10.1080/15502783.2022.2104130)
Supplement: Supplemental Material [file RSSN_A_2104130_SM1018.docx]

**Additional file 1**

**Table 1** Core domains of tendinopathy as defined by the ICON group (2019) (23)

| Domain | Description/definition | Example outcome |
| --- | --- | --- |
| Patient rating of condition | A single assessment numerical evaluation | Rate your tendon status where 100% is no problems and 0% worst case scenario, global rating of change, patient acceptable symptom status |
| Participating in life activities | Patient rating of the level of participating | Ratings of level of sport and time to return to sport |
| Pain on activity/loading | Patient reported intensity of pain on performing a task/activity that loads the tendon | VAS or NRS for pain intensity when the patient performs a tendon-specific pain-provocative task |
| Function | Patient rated level of function (and not referring to the intensity of their pain) | Patient Specific Function Scale on a VAS or NRS |
| Psychological factors | Psychology | Pain self-efficacy, pain catastrophisation, kinesiophobia, anxiety or depression scales |
| Physical function capacity | Quantitative measures of physical tasks performed in clinic | Number of hops, timed stair walk, number of single limb squats, including dynamometry and wearable technology |
| Disability | Composite scores of a mix of patient-rated pain and disability due to the pain, usually to tendon-specific activities/tasks | VISA scales, patient-rated tennis elbow evaluation, disability of the arm, shoulder and hand |
| Quality of life | The general well-being of the individual | Specific QoL questionnaires such as European QoL – 5 Dimension (EQ-5D) Australian QoL (AQoL), 36-item Short Form survey (SF-36) |
| Pain over a specified time | Participant reported pain intensity over a period of time (morning, night, 24 hours, a week) | VAS, NRS |
